# Performance Analysis of OCDM in ISAC Scenario

**DOI:** 10.3390/s25175481

**Published:** 2025-09-03

**Authors:** Pengfei Xu, Mao Li, Tao Zhan, Fengkui Gong, Yue Xiao, Xia Lei

**Affiliations:** 1 School of Telecommunications Engineering, Xidian University, Xi’an 710071, China; fkgong@xidian.edu.cn; 2 China Academy of Space Technology (Xi’an), Xi’an 710399, China; 3 National Key Laboratory of Science and Technology on Communications, University of Electronic Science and Technology of China, Chengdu 611731, China; limao@std.uestc.edu.cn (M.L.); ztzs1998@163.com (T.Z.); leixia@uestc.edu.cn (X.L.)

**Keywords:** Orthogonal Chirp Division Multiplexing (OCDM), Orthogonal Frequency Division Multiplexing (OFDM), Integrated Sensing and Communication (ISAC)

## Abstract

The rapid evolution of communication systems, exemplified by the Internet of Things (IoT), demands increasingly stringent reliability in both communication and sensing. While Orthogonal Frequency Division Multiplexing (OFDM) struggles to meet the challenges posed by complex scenarios, Orthogonal Chirp Division Multiplexing (OCDM) has gained attention for its robustness and spectral efficiency in Integrated Sensing and Communication (ISAC) systems. However, its sensing mechanism remains insufficiently explored. This paper presents a theoretical analysis of the communication and sensing performance of OCDM waveforms within the ISAC framework. Specifically, a closed-form BER expression under equalization is derived, alongside the ambiguity function and detection performance evaluation under matched filter (MF) and Generalized Likelihood Ratio Test (GLRT) detectors with a constant false alarm rate (CFAR) criterion. Simulation results demonstrate that OCDM offers comparable sensing performance to OFDM while achieving superior communication robustness in complex environments.

## 1. Introduction

Massive Machine-Type Communication (mMTC), exemplified by the Internet of Things (IoT), and Ultra-Reliable and Low-Latency Communication (uRLLC), typified by the Internet of Vehicles (IoV), constitute two of the three primary service categories envisioned for Fifth-Generation (5G) networks [1]. Looking ahead, Sixth-Generation (6G) systems have proposed six major application scenarios, including massive connectivity and Integrated Sensing and Communication (ISAC) [2,3]. These trends signal a convergence of communication and sensing functions, imposing more stringent requirements on waveform design [4,5,6,7,8,9]. Orthogonal Frequency Division Multiplexing (OFDM) has been widely adopted and extensively studied due to its high spectral efficiency and low-complexity equalization [10,11,12]. However, OFDM is prone to burst errors resulting from deep channel fades or external interference, which can severely degrade Bit Error Rate (BER) performance and render it inadequate for meeting the ultra-reliability demands of uRLLC. Simultaneously, mMTC scenarios also challenge the suitability of OFDM, as the operational frequency bands are highly susceptible to interference [13]. These limitations have motivated researchers to explore alternative waveform solutions, such as Orthogonal Chirp Division Multiplexing (OCDM).

OCDM is a recently proposed multi-carrier transmission scheme applicable to both optical fiber and wireless communication systems [14]. By employing chirp waveforms via the Discrete Fresnel Transform (DFnT), OCDM provides robust and secure link connectivity in complex electromagnetic environments, such as those encountered in IoT applications, due to its wide bandwidth occupancy. In an OCDM system, a set of orthogonal chirp waveforms is multiplexed, enabling high spectral efficiency while maintaining robustness and security, similar to OFDM. Prior studies have rigorously analyzed the communication performance of OCDM [15,16,17,18,19,20,21], demonstrating that OCDM achieves communication performance comparable to OFDM and can even attain a lower BER under certain fading channel conditions. These analyses include both simulation-based and theoretical evaluations, providing a balanced assessment of OCDM’s performance across diverse scenarios. The structural similarity between OCDM and OFDM also facilitates the study of OCDM-based ISAC systems. For example, Ref. [22] evaluates the sensing performance of OCDM waveforms through simulations and proposes a detector based on the Generalized Likelihood Ratio Test (GLRT). Moreover, Refs. [23,24,25] investigate Multiple Input Multiple Output (MIMO)-OCDM schemes for target velocity, range, and orientation sensing, showing performance comparable to MIMO-OFDM systems and providing insights into the theoretical limits of OCDM-based sensing. Additionally, Refs. [26,27,28] examine the integration of OCDM waveforms with radio resource management in ISAC systems. In particular, Ref. [26] introduces a quality-of-service-aware and channel-aware resource management framework that optimally allocates resources across heterogeneous services and parameter configurations in 5G networks.

However, the aforementioned studies do not offer a comprehensive analysis of the fundamental communication performance and sensing principles of OCDM waveforms. To address this gap, this work explores the communication and sensing performance of OCDM from a theoretical perspective. The key contributions are as follows:

- Derivation of the closed-form solution for the BER: A series of closed-form expressions of BER are derived for OCDM and related systems in the linear equalization from the perspective of precoded OFDM system, and the performance gains across these systems are analyzed.
- Derivation of the ambiguity function: The ambiguity functions of the OCDM and OFDM waveforms are derived to preliminarily evaluate their suitability for ISAC applications.
- Analysis of the detection performance of the OCDM waveform: The theoretical expressions for the detection probability of OCDM under a constant false alarm rate (CFAR) criterion using a matched filter (MF) and GLRT detector are formulated, with a corresponding training symbol design proposed.

The remainder of this paper is structured as follows. Section 2 introduces the OCDM system model. Section 3 analyzes the BER performance of OCDM with equalization. Section 4 derives the ambiguity function. Section 5 presents the detection performance of OCDM, with simulation results and comparisons detailed in Section 6. Finally, conclusions are drawn in Section 7. The abbreviations employed in this paper are summarized in Table 1.

## 2. OCDM System Model

OCDM is founded on the Fresnel transform, with chirp waveforms serving as its core building blocks. An OCDM system can be constructed using *N* orthogonal chirp waveforms, where the *k*-th subcarrier $\psi_{k}\left(t\right)$ is expressed as

(1) $$\psi_{k}\left(t\right)=e^{j\frac{\pi }{4}}e^{-j\pi \frac{N}{T^{2}}{\left(t-k\frac{T}{N}\right)}^{2}},0⩽t<T,$$

where k=0,…,N−1 denotes the subcarrier index, and *T* represents the signal duration. As shown in (1), the set of chirp signals is mutually orthogonal, which is further demonstrated in [29].

**Figure 1:**
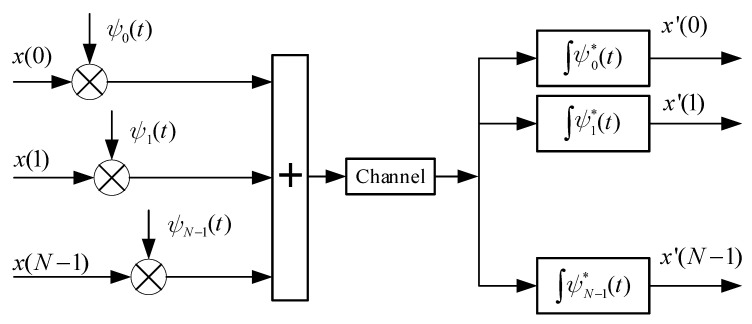
Schematic of the OCDM system.

Based on the orthogonal chirp waveforms, the transmitter maps parallel data symbols onto each subcarrier $\psi_{k}\left(t\right)$. At the receiver, these signals then can be processed using a matched filter, as illustrated in Figure 1. Let x(k) denote the symbol modulated onto the *k*-th chirp waveform, where k=0,1,…,N−1. Then, the transmitted signal is given by:

(2) $$s\left(t\right)=\sum_{k=0}^{N-1}x\left(k\right)\psi_{k}\left(t\right),0⩽t<T.$$

To recover the symbol transmitted on the *k*-th subcarrier, the receiver employs a matched filter defined as:

(3) $$\begin{array}{cc} x^{\prime }\left(m\right) & =\int_{0}^{T}s\left(t\right)\psi_{m}^{*}\left(t\right)dt=\sum_{k=0}^{N-1}x(k)\delta (m-k)=x\left(m\right). \end{array}$$

OCDM can be digitally implemented using the Inverse Discrete Fresnel Transform (IDFnT), in a manner analogous to the Fourier transform implementation of OFDM. To obtain the DFnT matrix, first consider sampling of the discrete-time OCDM signal s(n) in (2):

(4) $$\begin{array}{cc} \; & s\left(n\right)=\left\{\begin{array}{cc} s\left(t\right){|}_{t=n\frac{T}{N}} & N\equiv 0(\mathrm{mod}2)\\ s\left(t\right){|}_{t=\left(n+\frac{1}{2}\right)\frac{T}{N}} & N\equiv 1(\mathrm{mod}2) \end{array}\right.\\ \; & =e^{j\frac{\pi }{4}}\sum_{k=0}^{N-1}x\left(k\right)\times \left\{\begin{array}{cc} e^{-j\frac{\pi }{N}{\left(n-k\right)}^{2}} & N\equiv 0(\mathrm{mod}2)\\ e^{-j\frac{\pi }{N}{\left(n-k+\frac{1}{2}\right)}^{2}} & N\equiv 1(\mathrm{mod}2) \end{array}\right.. \end{array}$$

Thus, the element in the *m*-th row and *n*-th column of the DFnT matrix Φ is given by:

(5) $$\begin{array}{c} \Phi \left(m,n\right)=\frac{1}{\sqrt{N}}e^{-j\frac{\pi }{4}}\times \left\{\begin{array}{cc} e^{j\frac{\pi }{N}{\left(m-n\right)}^{2}} & N\equiv 0(\mathrm{mod}2)\\ e^{j\frac{\pi }{N}{\left(m-n+\frac{1}{2}\right)}^{2}} & N\equiv 1(\mathrm{mod}2) \end{array}\right.. \end{array}$$

From the above equation, it follows that the matrix Φ is unitary, and the IDFnT matrix corresponds to the conjugate transpose of the DFnT matrix. Consequently, the discrete OCDM signal can be obtained by applying the IDFnT to the constellation-mapped symbols. Therefore, the transmitted discrete OCDM signal can be expressed using Φ as:

(6) $$\begin{array}{c} s=\Phi^{H}x. \end{array}$$

To mitigate inter-block interference caused by multipath fading channels, a Cyclic Prefix (CP) of length $L_{cp}$ is appended to each OCDM block. To ensure proper signal recovery, the CP length must satisfy $L_{cp}\geq L_{m}$, where $L_{m}$ denotes the maximum delay spread of the channel. After transmission through the multipath fading channel and subsequent CP removal at the receiver, the received signal can be expressed as:

(7) $$\begin{array}{c} y=Hs+n=H\Phi^{H}x+n, \end{array}$$

where $H\in C^{N\times N}$ denotes a circulant matrix whose first column corresponds to the channel impulse response h. Although h is an *N*-dimensional vector, the energy of the wireless channel is typically concentrated in only a few paths. Thus, h can be represented as $h={\left[h_{1},h_{2},\ldots ,h_{K},0,\ldots ,0\right]}^{T}$, where $K\leq L_{m}$ denotes the number of significant multipath components. The noise vector $n\in C^{N}$ is modeled as complex Gaussian noise, i.e., $n∼\mathrm{CN}(0,\sigma^{2}I)$.

After signal reception, operations such as time-frequency synchronization, channel estimation, and equalization are necessary. In the following, we assume that time-frequency synchronization and channel estimation have been completed, and focus on the channel equalization process.

Applying DFnT to the signal in (7) yields

(8) $$\begin{array}{c} \Phi y=\Phi H\Phi^{H}x+\Phi n. \end{array}$$

Since H is a circulant matrix, it satisfies the following property based on the characteristics of DFnT matrices:

(9) $$\begin{array}{c} \Phi H\Phi^{H}=H_{diag}, \end{array}$$

where H is diagonalized as $H_{diag}$. Accordingly, (8) can be expressed as

(10) $$\begin{array}{c} \Phi y=H_{diag}x+\Phi n. \end{array}$$

From the above equation, it can be observed that after applying the DFnT at the receiver, the received signal in the OCDM system is equivalent to that of a conventional single-carrier system. Subsequently, applying the Discrete Fourier Transform (DFT) to this signal yields

(11) $$\begin{array}{cc} F\Phi y & ={\mathrm{FH}}_{diag}x+F\Phi n=FH_{diag}F^{H}Fx+F\Phi n=\Gamma \mathrm{Fx}+F\Phi n, \end{array}$$

where F is DFT matrix and $\Gamma =FH_{diag}F^{H}$ is a diagonal matrix. Let the equalization matrix be denoted as Λ. Applying Inverse Discrete Fourier Transform (IDFT) after equalization yields the estimated transmitted signal as

(12) $$\begin{array}{c} x^{\prime }=F^{H}\Lambda \Gamma \mathrm{Fx}+F^{H}\Lambda F\Phi n. \end{array}$$

If Zero Forcing (ZF) equalization is employed, then $\Lambda =\Gamma^{-1}$, and the above equation can be rewritten as

(13) $$\begin{array}{c} x^{\prime }=x+F^{H}\Gamma^{-1}F\Phi n. \end{array}$$

If Minimum Mean Square Error (MMSE) equalization is employed, then

(14) $$\begin{array}{c} \Lambda \left(k\right)=\frac{\Gamma^{*}\left(k\right)}{|\Gamma \left(k\right){|}^{2}+\sigma^{2}}, \end{array}$$

where Λ(k) denotes the *k*-th diagonal element of Λ. Thus, (12) can be expressed as

(15) $$\begin{array}{c} x^{\prime }=F^{H}\frac{\Gamma^{H}\Gamma }{\Gamma^{H}\Gamma +\sigma^{2}I}\mathrm{Fx}+F^{H}\frac{\Gamma^{H}}{\Gamma^{H}\Gamma +\sigma^{2}I}F\Phi n. \end{array}$$

The above derivation assumes a Linear Time-Invariant (LTI) channel. However, in the presence of time-varying channels with Doppler shifts, inter-carrier interference arises. To prevent excessive amplification of interference power caused by channel equalization at high Signal-to-Noise Ratios (SNRs), the following modification should be applied to (14):

(16) $$\begin{array}{c} \Lambda \left(k\right)=\frac{\Gamma^{*}\left(k\right)}{|\Gamma \left(k\right){|}^{2}+\sigma^{2}+\varepsilon }, \end{array}$$

where ε denotes the power of various disturbances [21].

## 3. BER Analysis of OCDM with Equalization

As shown by (1), in the frequency domain, the energy of each OCDM subcarrier is distributed across the entire system bandwidth, in contrast to OFDM, where the energy of each subcarrier is confined to a narrow frequency band, exhibiting frequency domain expansion characteristics of OCDM. This section quantitatively derives the BER of the OCDM system under linear equalization from the perspective of a precoded OFDM system, and analyzes the BER performance gain of the OCDM system based on this derivation.

### 3.1. ZF Equalization

Equation (6) can be equivalently expressed as

(17) $$\begin{array}{c} s=\Phi^{H}x=F^{H}F\Phi^{H}x. \end{array}$$

Let $G=F\Phi^{H}$, such that the OCDM system can be interpreted as an OFDM system with a precoding matrix G [30,31,32]. Moreover, $G^{H}G=I$, indicating that the precoding matrix G is unitary.

In fact, both the conventional OFDM system and Single-Carrier Frequency-Domain Equalization (SC-FDE) system can be interpreted as special cases of a precoded OFDM framework. Specifically, the OFDM system employs an identity matrix as its precoding matrix, whereas the SC-FDE system uses a DFT matrix F as the precoder. More generally, let the precoding matrix be denoted by G, where $G^{H}G=I$, indicating that G is unitary. Under this unified formulation, G serves as the general precoding matrix applicable to all three systems.

Let s denote the signal after Quadrature Amplitude Modulation (QAM). After precoding and OFDM modulation, the signal is transformed as:

(18) $$\begin{array}{c} s=F^{H}\mathrm{Gx}. \end{array}$$

After transmission through the multipath fading channel, a CP is added at the transmitter and subsequently removed at the receiver. The resulting signal becomes:

(19) $$\begin{array}{c} y=\mathrm{Hs}+n=HF^{H}\mathrm{Gx}+n. \end{array}$$

The recovered signal at the receiver, after applying the DFT, channel equalization, and decoding, is given by:

(20) $$\begin{array}{cc} x^{\prime } & =G^{H}\Lambda \mathrm{FH}F^{H}\mathrm{Gx}+G^{H}\Lambda \mathrm{Fn}=G^{H}\Lambda \Gamma \mathrm{Gx}+G^{H}\Lambda \mathrm{Fn}. \end{array}$$

where $\Gamma =FHF^{H}$ is diagonal, with its diagonal elements denoted by $\left[\Gamma_{0},\Gamma_{1},\ldots ,\Gamma_{N-1}\right]$. Let Λ represent the equalization matrix. Under ZF equalization, $\Lambda =\Gamma^{-1}$, and thus the above equation becomes:

(21) $$\begin{array}{c} x^{\prime }=x+G^{H}\Gamma^{-1}\mathrm{Fn}. \end{array}$$

Let the noise be defined as $q=\Gamma^{-1}Fn$. Since Fn and n share the same distribution, the variance of $q_{k}$ for k=0,1,…,N−1 is given by $\sigma_{q_{k}}^{2}=\sigma^{2}/{\left|\Gamma_{k}\right|}^{2}$. The output noise after decoding is $e=G^{H}q$, and the noise power on the *i*-th subcarrier is:

(22) $$\begin{array}{c} e_{i}=\sum_{k=0}^{N-1}g_{i,k}^{*}q_{k}, \end{array}$$

where $g_{i,k}$ denotes the element in the *k*-th row and *i*-th column of G, and ${\left(\cdot \right)}^{*}$ denotes the complex conjugate. Since the components $q_{k}$ are mutually independent, the noise variance on the *i*-th subcarrier is given by:

(23) $$\begin{array}{c} \sigma_{e}^{2}\left(i\right)=\sum_{k=0}^{N-1}|g_{i,k}{|}^{2}\sigma_{q_{k}}^{2}=\sigma^{2}\sum_{k=0}^{N-1}\frac{|g_{i,k}{|}^{2}}{|\Gamma_{k}{|}^{2}}. \end{array}$$

Let the signal power be denoted by $\varepsilon_{s}$. Then, the SNR of the received signal on the *i*-th subcarrier is expressed as:

(24) $$\begin{array}{c} \beta \left(i\right)=\frac{\varepsilon_{s}}{\sigma_{e}^{2}\left(i\right)}=\frac{\varepsilon_{s}/\sigma^{2}}{\sum_{k=0}^{N-1}\frac{|g_{i,k}{|}^{2}}{|\Gamma_{k}{|}^{2}}}=\frac{\gamma }{\sum_{k=0}^{N-1}\frac{|g_{i,k}{|}^{2}}{|\Gamma_{k}{|}^{2}}}, \end{array}$$

where $\gamma =\varepsilon_{s}/\sigma^{2}$ denotes the SNR of the transmitted signal. Under Binary Phase-Shift Keying (BPSK) or Quadrature Phase-Shift Keying (QPSK) modulation, the BER of the signal on the *i*-th subcarrier is given by:

(25) $$\begin{array}{c} p_{G}\left(i\right)=Q\left(\sqrt{\beta (i)}\right), \end{array}$$

where $Q\left(x\right)=1/\sqrt{2\pi }\int_{x}^{\infty }e^{-t^{2}/2}dt,x\geq 0$.

The average BER is

(26) $$\begin{array}{cc} p_{G} & =\frac{1}{N}\sum_{i=0}^{N-1}p_{G}\left(i\right)=\frac{1}{N}\sum_{i=0}^{N-1}Q\left(\sqrt{\beta (i)}\right)=\frac{1}{N}\sum_{i=0}^{N-1}Q\left(\sqrt{\frac{\gamma }{\sum_{k=0}^{N-1}\frac{|g_{i,k}{|}^{2}}{|\Gamma_{k}{|}^{2}}}}\right). \end{array}$$

For the OFDM system, the precoding matrix is G=I. Consequently, the noise variance on the *i*-th subcarrier is $\sigma_{e}^{2}\left(i\right)=\sigma^{2}/{\left|\Gamma_{i}\right|}^{2}$, and $\beta \left(i\right)=\gamma |\Gamma_{i}{|}^{2}$. Therefore, the average BER can be expressed as:

(27) $$\begin{array}{c} p_{\mathrm{OFDM}}=\frac{1}{N}\sum_{i=0}^{N-1}Q(\sqrt{\gamma |\Gamma_{i}{|}^{2}})=\frac{1}{N}\sum_{i=0}^{N-1}Q(\sqrt{\frac{1}{\frac{1}{\gamma |\Gamma_{i}{|}^{2}}}}). \end{array}$$

For the SC-FDE system, the precoding matrix is a DFT matrix, whose elements each have a magnitude of $1/\sqrt{N}$.

In fact, the precoding matrix of the OCDM system, $G=F\Phi^{H}$, also satisfies this condition. Without loss of generality, assuming the matrix order *N* is even, we have:

(28) $$\begin{array}{c} F\left(m,n\right)=\frac{1}{\sqrt{N}}e^{-j2\pi \frac{mn}{N}}, \end{array}$$

(29) $$\begin{array}{c} \Phi^{H}\left(m,n\right)=\frac{1}{\sqrt{N}}e^{j\frac{\pi }{4}}e^{-j\frac{\pi }{N}{\left(m-n\right)}^{2}}, \end{array}$$

where m,n=0,1,…,N−1; thus

(30) $$\begin{array}{cc} G(m,n) & =\sum_{k=0}^{N-1}F\left(m,k\right)\Phi^{H}\left(k,n\right)=\frac{1}{N}e^{j\frac{\pi }{4}}\sum_{k=0}^{N-1}e^{-j2\pi \frac{mk}{N}}e^{-j\pi \frac{{\left(k-n\right)}^{2}}{N}}\\ \; & =\frac{1}{N}e^{j\frac{\pi }{4}}e^{-j\pi \frac{n^{2}}{N}}e^{j\pi \frac{{\left(m-n\right)}^{2}}{N}}\sum_{k=0}^{N-1}e^{-j\pi \frac{{\left[k+(m-n)\right]}^{2}}{N}}. \end{array}$$

From the above equation, it is evident that the magnitudes of the elements of G depend solely on the summation term. This summation corresponds to a circular shift of the Zadoff–Chu (ZC) sequence $e^{-j\pi k^{2}/N}$ (k=0,1,…,N−1) by m−n positions. Since the summation involves the same ZC sequence regardless of the indices m,n, it is independent of these indices. Moreover, the magnitude of this sum equals $\sqrt{N}$, implying that each element of the precoding matrix G has a constant magnitude of $1/\sqrt{N}$.

Therefore, for OCDM and SC-FDE systems, there are:

(31) $$\begin{array}{c} \sigma_{e}^{2}\left(i\right)=\frac{\sigma^{2}}{N}\sum_{k=0}^{N-1}\frac{1}{|\Gamma_{k}{|}^{2}}, \end{array}$$

so the SNR on each subcarrier is given by

(32) $$\begin{array}{c} \beta \left(i\right)=\frac{\gamma }{\frac{1}{N}\sum_{k=0}^{N-1}\frac{1}{|\Gamma_{k}{|}^{2}}}=\frac{1}{\frac{1}{N}\sum_{k=0}^{N-1}\frac{1}{\gamma |\Gamma_{k}{|}^{2}}}. \end{array}$$

The above equation indicates that the output SNR on each subcarrier of both the OCDM and SC-FDE systems is identical. Consequently, the average BER is expressed as

(33) $$\begin{array}{c} p_{\mathrm{OCDM}(\mathrm{SC}-\mathrm{FDE})}=Q\left(\sqrt{\frac{1}{\frac{1}{N}\sum_{k=0}^{N-1}\frac{1}{\gamma |\Gamma_{k}{|}^{2}}}}\right) \end{array}$$

Define the function

(34) $$\begin{array}{c} f\left(y\right)=Q\left(1/\sqrt{y}\right). \end{array}$$

The difference between (27) and (33) is that OFDM averages the values of f(y) across subcarriers, whereas OCDM and SC-FDE apply f(y) to the average SNR across subcarriers. This distinction arises from the convexity properties of the function f(y). Therefore, to further analyze the BER performance of conventional OFDM, SC-FDE, and OCDM systems under BPSK and QPSK modulation, this paper presents the following theorem regarding the convexity of f(y).

**Theorem 1.**  *f(y) is convex for 0<y≤1/3 and concave for y>1/3.*

**Proof of Theorem** **1.**  Let $u\left(y\right)=\frac{1}{\sqrt{y}}$; hence, its first and second derivatives are $u^{\prime }\left(y\right)=-\frac{1}{2}y^{-3/2}$ and $u^{\prime \prime }\left(y\right)=\frac{3}{4}y^{-5/2}$, respectively. Define f(u)=Q(u), with derivatives $f^{\prime }\left(u\right)=-\frac{1}{\sqrt{2\pi }}e^{-u^{2}/2}$ and $f^{\prime \prime }\left(u\right)=\frac{u}{\sqrt{2\pi }}e^{-u^{2}/2}$. Then, by applying the chain rule, we have:

(35) $$\begin{array}{c} f^{\prime }\left(u\left(y\right)\right)=f^{\prime }\left(u\right)u^{\prime }\left(y\right)=\frac{1}{2\sqrt{2\pi }}y^{-\frac{3}{2}}e^{-\frac{1}{2y}}. \end{array}$$

For y>0, we have $f^{\prime }\left(u\left(y\right)\right)>0$, indicating that f(y) is monotonically increasing in this domain. Moreover, it can be observed that

(36) $$\begin{array}{cc} f^{\prime \prime }\left(u\left(y\right)\right) & =f^{\prime \prime }\left(u\right){\left[u^{\prime }\left(y\right)\right]}^{2}+f^{\prime }\left(u\right)u^{\prime \prime }\left(y\right)=\frac{1}{4\sqrt{2\pi }}e^{-\frac{1}{2y}}y^{-\frac{7}{2}}-\frac{3}{4\sqrt{2\pi }}e^{-\frac{1}{2y}}y^{-\frac{5}{2}}\\ \; & =\frac{1}{4\sqrt{2\pi }}e^{-\frac{1}{2y}}y^{-\frac{5}{2}}\left(y^{-1}-3\right). \end{array}$$

Therefore, when $f^{\prime \prime }\left(u\left(y\right)\right)\geq 0$, it follows that $0<y\leq \frac{1}{3}$; conversely, when $f^{\prime \prime }\left(u\left(y\right)\right)<0$, it holds that $y>\frac{1}{3}$. Hence, Theorem 1 is proved. □

To analyze the BER performance of the three systems under varying SNR conditions, it is also necessary to examine the SNR relationships among them. To this end, the following theorem is presented in this paper.

**Theorem 2.**  *For any OFDM system employing unitary matrix precoding, the SNR on the i-th subcarrier satisfies the following relationship:*

(37) $$\begin{array}{c} \min_{k}\beta_{\mathrm{OFDM}}\left(k\right)\leq \beta \left(i\right)\leq \max_{k}\beta_{\mathrm{OFDM}}\left(k\right). \end{array}$$

*In other words, for OFDM systems employing unitary matrix precoding, the SNR of any subcarrier at the receiver is upper-bounded by the maximum SNR and lower-bounded by the minimum SNR among all subcarriers in a conventional OFDM system.*

**Proof of Theorem** **2.**  From (27) and (31), the following expression is obtained:

(38) $$\begin{array}{c} \sigma_{e}^{2}\left(i\right)=\sum_{k=0}^{N-1}|g_{i,k}{|}^{2}\sigma_{\mathrm{OFDM}}^{2}\left(k\right). \end{array}$$

Since $\sum_{k=0}^{N-1}|g_{i,k}{|}^{2}=1$, it follows that:

(39) $$\begin{array}{c} \sigma_{e}^{2}\left(i\right)\leq \sum_{k=0}^{N-1}|g_{i,k}{|}^{2}\max_{k}\sigma_{\mathrm{OFDM}}^{2}\left(k\right)=\max_{k}\sigma_{\mathrm{OFDM}}^{2}\left(k\right). \end{array}$$

The same reasoning applies to the other inequality, so Theorem 2 holds. □

Define

(40) $$\begin{array}{c} \gamma_{0}=\min_{i}\frac{3}{|\Gamma_{i}{|}^{2}},\gamma_{1}=\max_{i}\frac{3}{|\Gamma_{i}{|}^{2}}. \end{array}$$

When $\gamma \leq \gamma_{0}$, the output SNR of all subcarriers in both the OCDM and SC-FDE systems satisfies β(i)≤3. According to Theorem 1, the corresponding BER lies within the concave region of the function f(y). Therefore, the following inequality holds: $p_{\mathrm{OFDM}}\leq p_{\mathrm{OCDM}(\mathrm{SC}\text{-}\mathrm{FDE})}$.

When $\gamma >\gamma_{1}$, the output SNR of all subcarriers in the OCDM and SC-FDE systems satisfies β(i)>3. According to Theorem 1, the corresponding BER lies within the convex region of the function f(y). Consequently, the following inequality holds: $p_{\mathrm{OFDM}}>p_{\mathrm{OCDM}(\mathrm{SC}\text{-}\mathrm{FDE})}$.

The above conclusions indicate that, under ZF equalization and BPSK (or QPSK) modulation, conventional OFDM outperforms any unitarily precoded OFDM variant—including OCDM and SC-FDE—at low SNRs. Conversely, OCDM and SC-FDE outperform conventional OFDM at high SNRs. The intersection of their BER curves corresponds to an SNR value near $\bar{\gamma }$, which satisfies:

(41) $$\begin{array}{c} \bar{\gamma }=\frac{1}{N}\sum_{i=0}^{N-1}\frac{3}{|\Gamma_{i}{|}^{2}}. \end{array}$$

It is worth noting that although the above conclusions are derived for BPSK (or QPSK) modulation, similar trends hold for higher-order modulation schemes. However, the corresponding threshold values $\gamma_{0}$ and $\gamma_{1}$ increase accordingly.

For the complexity of ZF equalization, according to (20), it can be decomposed into three components: the DFT transformation, the multiplication with the diagonal equalization matrix, and the multiplication with the precoding matrix. For all three systems, the DFT operation can be efficiently implemented using the Fast Fourier Transform (FFT) algorithm, which yields a complexity of O(NlogN). The multiplication with the diagonal matrix Γ has a complexity of O(N). In the case of the OFDM system, since G=I, there is no additional computational overhead. For the SC-FDE system, G=F, and thus the complexity of the precoding matrix multiplication corresponds to that of an inverse DFT, namely O(NlogN). For the OCDM system, $G=F\Phi^{H}$, which requires one inverse DFT and one DFnT operation. The DFnT can be implemented using two FFTs and one frequency-domain multiplication, resulting in a total complexity of O(3NlogN+N). In summary, the complexity of OFDM is O(NlogN+N), the complexity of SC-FDE is O(2NlogN+N), and the complexity of OCDM is O(4NlogN+2N). All three are O(NlogN)-level.

### 3.2. MMSE Equalization

Under MMSE equalization, the equalization matrix satisfies:

(42) $$\begin{array}{c} \Lambda =\mathrm{diag}(\lambda_{0},\lambda_{1},\ldots ,\lambda_{N-1}), \end{array}$$

where $\lambda_{k}=\gamma \Gamma_{k}^{*}/\left(1+\gamma |\Gamma_{k}{|}^{2}\right)$. The signal recovered on the *i*-th subcarrier is then given by:

(43) $$\begin{array}{c} x_{i}^{\prime }=a_{i}x_{i}+\tau_{i}, \end{array}$$

where

(44) $$\begin{array}{c} a_{i}=\sum_{k=0}^{N-1}|g_{i,k}{|}^{2}\frac{\gamma |\Gamma_{k}{|}^{2}}{1+\gamma |\Gamma_{k}{|}^{2}} \end{array}$$

and

(45) $$\begin{array}{c} \tau_{i}=\sum_{j=0,j\neq i}^{N-1}x_{j}\sum_{k=0}^{N-1}\left(g_{i,k}^{*}g_{j,k}\frac{\gamma |\Gamma_{k}{|}^{2}}{1+\gamma |\Gamma_{k}{|}^{2}}\right)+{\left[G^{H}\Lambda \mathrm{Fn}\right]}_{i}. \end{array}$$

In the above equation, $\tau_{i}$ denotes the noise component on the *i*-th subcarrier. The noise power on the *i*-th subcarrier is given by $E[|\tau_{i}{|}^{2}]$, while the corresponding signal power is $a_{i}^{2}\varepsilon_{s}$. Accordingly, the SNR of the output signal on the *i*-th subcarrier is:

(46) $$\begin{array}{c} \beta \left(i\right)=\frac{a_{i}^{2}\varepsilon_{s}}{E[|\tau_{i}{|}^{2}]}. \end{array}$$

Since different signal symbols and noise components are mutually independent, it follows that:

(47) $$\begin{array}{c} \beta \left(i\right)=\frac{\sum_{k=0}^{N-1}\frac{|g_{i,k}{|}^{2}\gamma |\Gamma_{k}{|}^{2}}{1+\gamma |\Gamma_{k}{|}^{2}}}{\sum_{k=0}^{N-1}\frac{|g_{i,k}{|}^{2}}{1+\gamma |\Gamma_{k}{|}^{2}}}. \end{array}$$

Therefore, the BER of the output signal on the *i*-th subcarrier is given by $p\left(i\right)=Q\left(\sqrt{\beta (i)}\right)$.

Let

(48) $$\begin{array}{c} h\left(y\right)=Q\left(\sqrt{1/y-1}\right). \end{array}$$

A corresponding theorem regarding the convexity of functions, analogous to Theorem 1, can also be formulated.

**Theorem 3.**  *The function h(y) is monotonically increasing and convex over the interval 0<y<1.*

**Proof of Theorem** **3.**  Let $u\left(y\right)=\sqrt{1/y-1}$, then

(49) $$\begin{array}{c} u^{\prime }\left(y\right)=-\frac{1}{2}{\left(1-y\right)}^{-\frac{1}{2}}y^{-\frac{1}{2}}-\frac{1}{2}{\left(1-y\right)}^{\frac{1}{2}}y^{-\frac{3}{2}} \end{array}$$

and

(50) $$\begin{array}{c} u^{\prime \prime }\left(y\right)=\frac{3}{4}{\left(1-y\right)}^{\frac{1}{2}}y^{-\frac{5}{2}}-\frac{1}{4}{\left(1-y\right)}^{-\frac{3}{2}}y^{-\frac{1}{2}} \end{array}$$

Thus

(51) $$\begin{array}{cc} \; & h^{\prime }\left(u\left(y\right)\right)=h^{\prime }\left(u\right)u^{\prime }\left(y\right)=\frac{1}{2}\sqrt{2\pi }e^{-\frac{u^{2}\left(y\right)}{2}}\left[{\left(1-y\right)}^{-\frac{1}{2}}y^{-\frac{1}{2}}+{\left(1-y\right)}^{\frac{1}{2}}y^{-\frac{3}{2}}\right]. \end{array}$$

When 0<y<1, it follows that $h^{\prime }\left(u\left(y\right)\right)>0$, indicating that h(y) is monotonically increasing over this interval. Furthermore, it can be observed that

(52) $$\begin{array}{cc} \; & h^{\prime \prime }\left(u\left(y\right)\right)=h^{\prime \prime }\left(u\right){\left[u^{\prime }\left(y\right)\right]}^{2}+h^{\prime }\left(u\right)u^{\prime \prime }\left(y\right)\\ \; & =\frac{u(y)}{\sqrt{2\pi }}e^{-\frac{u^{2}\left(y\right)}{2}}{\left[u^{\prime }\left(y\right)\right]}^{2}-\frac{1}{\sqrt{2\pi }}e^{-\frac{u^{2}\left(y\right)}{2}}\left[\frac{3}{4}{\left(1-y\right)}^{\frac{1}{2}}y^{-\frac{5}{2}}-\frac{1}{4}{\left(1-y\right)}^{-\frac{3}{2}}y^{-\frac{1}{2}}\right]. \end{array}$$

It follows directly that

(53) $$\begin{array}{c} \frac{3}{4}{\left(1-y\right)}^{\frac{1}{2}}y^{-\frac{5}{2}}<\frac{1}{4}{\left(1-y\right)}^{-\frac{3}{2}}y^{-\frac{1}{2}} \end{array}$$

holds for 0<y<1, implying that $h^{\prime \prime }\left(u\left(y\right)\right)>0$. Therefore, h(y) is a convex function over the interval 0<y<1, which confirms the validity of Theorem 3. □

Let β(i)=1/y−1, which implies y=1/(β(i)+1). Thus

(54) $$\begin{array}{c} p\left(i\right)=Q\left(\sqrt{\beta (i)}\right)=h\left(\frac{1}{\beta (i)+1}\right). \end{array}$$

From (47), it follows that:

(55) $$\begin{array}{cc} \frac{1}{\beta (i)+1} & =\frac{\sum_{k=0}^{N-1}\frac{|g_{i,k}{|}^{2}}{1+\gamma |\Gamma_{k}{|}^{2}}}{\sum_{k=0}^{N-1}\frac{|g_{i,k}{|}^{2}\gamma |\Gamma_{k}{|}^{2}}{1+\gamma |\Gamma_{k}{|}^{2}}+\frac{|g_{i,k}{|}^{2}}{1+\gamma |\Gamma_{k}{|}^{2}}}=\frac{\sum_{k=0}^{N-1}\frac{|g_{i,k}{|}^{2}}{1+\gamma |\Gamma_{k}{|}^{2}}}{\sum_{k=0}^{N-1}|g_{i,k}{|}^{2}}=\sum_{k=0}^{N-1}\frac{|g_{i,k}{|}^{2}}{1+\gamma |\Gamma_{k}{|}^{2}}. \end{array}$$

For the OCDM and SC-FDE systems, it holds that $|g_{i,k}{|}^{2}=1/N$; therefore

(56) $$\begin{array}{c} p_{\mathrm{OCDM}(\mathrm{SC}-\mathrm{FDE})}=p\left(i\right)=h\left(\frac{1}{N}\sum_{k=0}^{N-1}\frac{1}{1+\gamma |\Gamma_{k}{|}^{2}}\right). \end{array}$$

For traditional OFDM systems, it holds that

(57) $$\begin{array}{c} |g_{i,k}{|}^{2}=\left\{\begin{array}{cc} 1 & k=i\\ 0 & k\neq i \end{array}\right.. \end{array}$$

Thus

(58) $$\begin{array}{c} p_{\mathrm{OFDM}}=\frac{1}{N}\sum_{i=0}^{N}p(i)=\frac{1}{N}\sum_{i=0}^{N-1}h\left(\frac{1}{1+\gamma |\Gamma_{i}{|}^{2}}\right). \end{array}$$

According to Theorem 3, $p_{\mathrm{OCDM}(\mathrm{SC}-\mathrm{FDE})}<p_{\mathrm{OFDM}}$ holds for all SNR values. Furthermore, by examining (27) and (58), it can be observed that the BER performance of the ZF and MMSE equalizers in OFDM systems is equivalent.

The above conclusions indicate that under MMSE equalization with BPSK (or QPSK) modulation, the BER performance of OCDM and SC-FDE systems surpasses that of conventional OFDM systems. For higher-order modulation, such as 16QAM, a similar trend to that observed under ZF equalization re-emerges: at low SNRs, the BER performance of OCDM (or SC-FDE) systems is inferior to that of OFDM, whereas at high SNRs, the situation reverses. However, compared to ZF equalization, the crossover point occurs at a lower SNR.

For the complexity of MMSE equalization, the primary difference from ZF lies in the structure of the equalization matrix Γ. However, the computational cost of determining all elements of the diagonal matrix Γ remains on the order of O(N), identical to that of ZF. Consequently, from the perspective of overall computational complexity, MMSE exhibits the same order as ZF.

### 3.3. Multipath Diversity Gain Analysis

The preceding sections analyzed the BER performance of the considered systems under two linear equalization methods by examining the SNR of each subcarrier’s output at the receiver. However, the underlying cause of the observed variations in the SNR remains unclear. The following section investigates multipath diversity gain to clarify the similarities and differences in BER performance among OFDM, OCDM, and SC-FDE.

As shown in (19), the received signal is given by:

(59) $$\begin{array}{cc} \; & r=HF^{H}\mathrm{Gx}+n=F^{H}\Gamma FF^{H}\mathrm{Gx}+n=F^{H}\Gamma \mathrm{Gx}+n, \end{array}$$

where $\Gamma =FHF^{H}$ denotes a diagonal matrix. Define the pairwise error event, when the actual transmitted signal is x, as $\left\{x\to x^{\prime }\right\}$, with $x\neq x^{\prime }$. The pairwise error probability (PEP) then satisfies:

(60) $$\begin{array}{c} P\left(x\to x^{\prime }|h\right)\leq e^{-\frac{d^{2}\left(r^{\prime },r\right)}{4\sigma^{2}}}, \end{array}$$

where $r^{\prime }=F^{H}\Gamma Gx^{\prime }+n$, and the squared distance is defined as $d^{2}\left(r^{\prime },r\right)=∥F^{H}\Gamma G\left(x^{\prime }-x\right){∥}^{2}={∥\Gamma G\left(x^{\prime }-x\right)∥}^{2}$. Let $e=G(x^{\prime }-x)$. Due to the diagonal structure of Γ, it follows that

(61) $$\begin{array}{c} d^{2}\left(r^{\prime },r\right)=\left||\Gamma e\right|{|}^{2}=\left|\right|D_{e}\tilde{h}|{|}^{2}, \end{array}$$

where $D_{e}=\mathrm{diag}\left(e\right)$ and $\tilde{h}={\left[\Gamma_{0},\Gamma_{1},\ldots ,\Gamma_{N-1}\right]}^{T}$ denote the channel frequency-domain response coefficients. Let $h={\left[h_{1},h_{2},\ldots ,h_{K}\right]}^{T}$. It then follows that:

(62) $$\begin{array}{c} \tilde{h}=F_{N\times K}h, \end{array}$$

where $F_{N\times K}$ denotes the matrix formed by the first *K* columns of the DFT matrix.

Let the channel correlation matrix $R_{h}=E\left(hh^{H}\right)$ be full rank. It can be decomposed as $R_{h}=BB^{H}$, where $B=R_{h}^{1/2}$ denotes the positive definite square root of $R_{h}$. Define the whitened channel vector $\bar{h}={\left[{\bar{h}}_{1},\ldots ,{\bar{h}}_{K}\right]}^{T}=B^{-1}h$, whose elements are independent and identically distributed to CN(0,1). Thus, (61) can be rewritten as:

(63) $$\begin{array}{c} d^{2}\left(r^{\prime },r\right)=\left|\right|D_{e}\tilde{h}|{|}^{2}=h^{H}A_{e}h={\bar{h}}^{H}C_{e}\bar{h}, \end{array}$$

where $A_{e}=F_{N\times K}^{H}D_{e}^{H}D_{e}F_{N\times K}$ and $C_{e}=B^{H}A_{e}B$. Considering all possible random channel realizations, we focus on the average PEP. For the random variables ${\bar{h}}_{l}$(l=1,…,K), (60) can be rewritten as:

(64) $$\begin{array}{c} P\left(x\to x^{\prime }\right)\leq \prod_{l=1}^{R(C_{e})}{\left(1+\frac{\lambda_{l}}{4\sigma^{2}}\right)}^{-1}, \end{array}$$

where $\lambda_{l}$ denotes the non-zero eigenvalues of $C_{e}$, and $R(C_{e})$ represents the rank of $C_{e}$.

Observing (64), it is evident that the upper bound of the average PEP decreases as $R(C_{e})$ increases. Therefore, $R(C_{e})$ can be employed to characterize the lower bound of the system’s error performance. Given that $C_{e}=B^{H}F_{N\times K}^{H}D_{e}^{H}D_{e}F_{N\times K}B$ with B being full rank, we define the multipath diversity gain as

(65) $$\begin{array}{c} Gain=R\left(C_{e}\right)=\min_{e\neq 0}\left(L,K\right), \end{array}$$

where *K* denotes the number of multipath components, and *L* represents the number of non-zero elements in e.

For OFDM systems, $e=G\left(x^{\prime }-x\right)=x^{\prime }-x$, implying that for any transmitted data x, the value of *L* may be as small as 1. Consequently, the achievable diversity gain of OFDM systems is limited to 1. In contrast, for OCDM and SC-FDE systems, $e=G(x^{\prime }-x)$, where G is a unitary matrix whose elements have equal modulus. Given that x takes values from a finite alphabet, the probability that L=1 is significantly reduced. In fact, this probability decreases exponentially with the number of subcarriers, as shown in [32].

The above analysis confirms that OCDM and SC-FDE systems can exploit multipath diversity; however, the extent of gain depends on the equalization strategy. As shown previously, the BER performance of OCDM (or SC-FDE) systems under ZF and MMSE equalization is constrained by an SNR threshold, regardless of the modulation scheme. Nonlinear equalization methods, such as Maximum Likelihood Equalization (MLE), better leverage this diversity but are computationally intensive and impractical for real-time implementation. Assume the modulation order is *M*. For the OFDM system, after applying the FFT, maximum likelihood detection across all subcarriers incurs a computational complexity of O(NM). For the SC-FDE system, due to temporal correlation among symbols, detection requires an exhaustive search over all possible time-domain symbol sequences, leading to a complexity of $O(M^{N}N^{2})$. Similarly, for the OCDM system, an exhaustive search over all possible symbol combinations across subcarriers is required, resulting in the same complexity order of $O(M^{N}N^{2})$. Even when employing approximate algorithms such as sphere decoding, the complexity still scales up to $O(N^{3})$.

Alternatively, Iterative Block-Decision Feedback Equalization (IB-DFE) achieves a favorable balance between complexity and performance. An overview of the IB-DFE principle is presented below [33]. Decision feedback equalization involves re-modulating the estimated information bits obtained after the initial demodulation stage, followed by the design of a feedback matrix based on the principle of minimizing the mean square error between the re-modulated constellation symbols and the pre-demodulation signal. Taking the OCDM system as an example, the decision feedback equalization filter is given by $M^{H}-I$, and the output of the decision feedback equalizer can be expressed as:

(66) $$\begin{array}{c} z=\hat{x}+\left(M^{H}-I\right)\left(\hat{x}-x\right), \end{array}$$

where M denotes the decision feedback matrix, which is a circulant matrix whose first column is given by:

(67) $$\begin{array}{cc} m_{1} & ={\left[1,m_{1},\ldots ,m_{N-1}\right]}^{T}={\left[1,\frac{h_{2}^{H}h_{1}}{h_{1}^{H}h_{1}+\sigma^{2}},\ldots ,\frac{h_{N}^{H}h_{1}}{h_{1}^{H}h_{1}+\sigma^{2}}\right]}^{T}\\ \; & =\frac{1}{h_{1}^{H}h_{1}+\sigma^{2}}{\left(H^{H}H+\sigma^{2}I\right)}_{:,1}, \end{array}$$

where $h_{i}$ (i=1,2,…,N) denotes the *i*-th column of the circulant matrix H, and ${\left(\cdot \right)}_{:,1}$ represents the first column of a matrix. The feedback matrix is derived by minimizing the mean square error between x and its estimate $\hat{x}$.

The computational complexity of IB-DFE is primarily attributed to the decision feedback stage. The matrix M can be efficiently computed by exploiting its circulant structure, which results in a complexity of $O(N^{2})$. Furthermore, the multiplications required for generating the feedback output also incur a complexity of $O(N^{2})$. Therefore, the overall complexity of IB-DFE is in the order of $O(N^{2})$. The complexity of each method is summarized in the Table 2.

## 4. Ambiguity Function Characteristic Analysis

The ambiguity function is a fundamental tool for evaluating the performance of sensing waveforms. Based on its characteristics, it can be used to analyze the sensitivity of a waveform to time delay and Doppler shift. A waveform whose ambiguity function magnitude decays more rapidly with increasing delay and Doppler exhibits superior resolution capability. The ambiguity function is defined as follows [34]:

(68) $$\begin{array}{c} \chi \left(\tau ,f_{d}\right)=\int_{-\infty }^{+\infty }u\left(t\right)u^{*}\left(t+\tau \right)e^{j2\pi f_{d}t}dt, \end{array}$$

where u(t) represents the envelope of the sensing transmission waveform, while τ and $f_{d}$ represent the time delay and Doppler frequency shift, respectively.

### 4.1. OFDM Ambiguity Function

Assume that $N_{s}$ OFDM symbols are transmitted within a single sensing pulse period, where each OFDM symbol contains $N_{c}$ subcarriers. Let *T* denote the duration of each subcarrier waveform, and ignore the cyclic prefix for simplicity. Then the transmitted signal can be expressed as

(69) $$\begin{array}{c} u\left(t\right)=\sum_{n=0}^{N_{s}-1}\sum_{m=0}^{N_{c}-1}a_{m,n}e^{j2\pi m\Delta ft}\mathrm{rect}\left[\frac{t-nT}{T}\right], \end{array}$$

where $a_{m,n}$ denotes the QAM symbol transmitted on the (m+1)-th subcarrier of the (n+1)-th OCDM symbol. The function rect[t] is defined as follows:

(70) $$\begin{array}{c} \mathrm{rect}\left[t\right]=\left\{\begin{array}{cc} 1 & 0<t\leq 1\\ 0 & \mathrm{others} \end{array}\right.. \end{array}$$

Substituting the above equation into (68) yields:

(71) $$\begin{array}{cc} \chi (\tau ,f_{d}) & =\int_{\infty }^{+\infty }u\left(t\right)u^{*}\left(t+\tau \right)e^{j2\pi f_{d}t}dt\\ \; & =\sum_{n=0}^{N_{s}-1}\sum_{m=0}^{N_{c}-1}\sum_{q=0}^{N_{s}-1}\sum_{p=0}^{N_{c}-1}a_{m,n}a_{p,q}^{*}e^{j2\pi p\Delta f\tau }\int_{a}^{b}e^{j2\pi [\left(m-p\right)\Delta f+f_{d}]t}dt. \end{array}$$

Let

(72) $$\begin{array}{cc} C(\left(m-p\right)\Delta f+f_{d}) & =\int_{a}^{b}e^{j2\pi [\left(m-p\right)\Delta f+f_{d}]t}dt\\ \; & =\left(b-a\right)e^{j\pi [\left(m-p\right)\Delta f+f_{d}]\left(a+b\right)}\mathrm{sinc}\left(\pi \left(\left(m-p\right)\Delta f+f_{d}\right)\left(b-a\right)\right), \end{array}$$

where sinc(x)=sin(x)/x. Since the range of *t* in rect[t] of u(t) is nT<t≤(n+1)T, and in rect[t] of $u^{*}\left(t+\tau \right)$ is qT−τ<t≤(q+1)T−τ, the integration limits are determined by a=max{nT,qT−τ} and b=min{(n+1)T,(q+1)T−τ}.

Furthermore, if we assume

(73) $$\begin{array}{c} \sum_{m,n,p,q}\left(\cdot \right)=\sum_{n=0}^{N_{s}-1}\sum_{m=0}^{N_{c}-1}\sum_{q=0}^{N_{s}-1}\sum_{p=0}^{N_{c}-1}\left(\cdot \right), \end{array}$$

and

(74) $$\begin{array}{cc} D(m,n,p,q & ,\tau ,f_{d})=a_{m,n}a_{p,q}^{*}e^{j2\pi p\Delta f\tau }C\left(\left(m-p\right)\Delta f+f_{d}\right), \end{array}$$

then

(75) $$\begin{array}{cc} \; & \chi \left(\tau ,f_{d}\right)=\sum_{m,n,p,q}D\left(m,n,p,q,\tau ,f_{d}\right). \end{array}$$

Therefore, the expression for the ambiguity function of the OFDM is presented as

(76) $$\left|\chi (\tau ,f_{d})\right|=\left\{\begin{array}{cc} \left|\sum_{m,n,p,q}D\left(m,n,p,q,\tau ,f_{d}\right)\right|, & |\tau |<N_{s}T,\\ 0, & \mathrm{others}, \end{array}\right.$$

When τ=0, the velocity ambiguity function is given by

(77) $$\begin{array}{c} \left|\chi (0,f_{d})\right|=\left|\sum_{m,n,p,q}D\left(m,n,p,q,0,f_{d}\right)\right|. \end{array}$$

And when $f_{d}=0$, the range ambiguity function is obtained as

(78) $$\left|\chi (\tau ,0)\right|=\left\{\begin{array}{cc} \left|\sum_{m,n,p,q}D\left(m,n,p,q,\tau ,0\right)\right|, & |\tau |<N_{s}T,\\ 0, & \mathrm{others}, \end{array}\right.$$

Subsequent simulations indicate that the OFDM-ISAC waveform exhibits sensitivity to both time delay and Doppler shift, thereby demonstrating its suitability for sensing applications.

### 4.2. OCDM Ambiguity Function

Similar with OFDM, assume that $N_{s}$ OCDM symbols are transmitted within a single sensing pulse period, where each OCDM symbol contains $N_{c}$ subcarriers. Let *T* denote the duration of each Chirp waveform, and ignore the cyclic prefix for simplicity. According to (1), the transmitted signal is given by:

(79) $$\begin{array}{c} u\left(t\right)=\sum_{n=0}^{N_{s}-1}\sum_{m=0}^{N_{c}-1}b_{m,n}e^{j\frac{\pi }{4}}e^{-j\pi \frac{N_{c}}{T^{2}}{\left(t-m\frac{T}{N_{c}}\right)}^{2}}\mathrm{rect}\left[\frac{t-nT}{T}\right], \end{array}$$

where $b_{m,n}$ denotes the data symbol transmitted on the *m*-th subcarrier of the *n*-th OCDM symbol. Substituting the above equation into (68) yields:

(80) $$\begin{array}{cc} \chi (\tau ,f_{d}) & =\int_{\infty }^{+\infty }u\left(t\right)u^{*}\left(t+\tau \right)e^{j2\pi f_{d}t}dt\\ \; & =\sum_{m,n,p,q}b_{m,n}b_{p,q}^{*}e^{j\pi \frac{N_{c}}{T^{2}}[\tau -\left(m+p\right)\frac{T}{N_{c}}][\tau +\left(m-p\right)\frac{T}{N_{c}}]}\int_{a}^{b}e^{j2\pi (\frac{m-p}{T}+\tau +f_{d})t}dt. \end{array}$$

Similar to (71), the integration limits are determined by a=max{nT,qT−τ} and b=min{(n+1)T,(q+1)T−τ}. Furthermore, let

(81) $$\begin{array}{cc} R & \left(\frac{m-p}{T}+\tau +f_{d}\right)=\int_{a}^{b}e^{j2\pi (\frac{m-p}{T}+\tau +f_{d})t}dt\\ \; & =\left(b-a\right)e^{j\pi \left(\frac{m-p}{T}+\tau +f_{d}\right)\left(a+b\right)}\mathrm{sinc}\left(\pi \left(\frac{m-p}{T}+\tau +f_{d}\right)\left(b-a\right)\right), \end{array}$$

(82) $$\begin{array}{c} e^{j\pi \frac{N_{c}}{T^{2}}[\tau -\left(m+p\right)\frac{T}{N_{c}}][\tau +\left(m-p\right)\frac{T}{N_{c}}]}=e^{j\pi \tau \frac{N_{c}}{T^{2}}\left(\tau -2p\frac{T}{N_{c}}\right)}e^{j\pi \frac{m^{2}-p^{2}}{N_{c}}}=E_{\tau }E_{t}, \end{array}$$

where $E_{\tau }=\exp\left(j\pi \tau N_{c}/T^{2}\left(\tau -2pT/N_{c}\right)\right)$ and $E_{t}=\exp\left(j\pi \left(m^{2}-p^{2}\right)/N_{c}\right)$. Let

(83) $$\begin{array}{cc} U(m,n,p,q & ,\tau ,f_{d})=b_{m,n}b_{p,q}^{*}E_{\tau }E_{t}R\left(\frac{m-p}{T}+\tau +f_{d}\right). \end{array}$$

Therefore, the expression for the ambiguity function of the OCDM is presented as

(84) $$\left|\chi (\tau ,f_{d})\right|=\left\{\begin{array}{cc} \left|\sum_{m,n,p,q}U\left(m,n,p,q,\tau ,f_{d}\right)\right|, & |\tau |<N_{s}T,\\ 0, & \mathrm{others}, \end{array}\right.$$

When τ=0, the velocity ambiguity function is given by

(85) $$\begin{array}{c} \left|\chi (0,f_{d})\right|=\left|\sum_{m,n,p,q}U\left(m,n,p,q,0,f_{d}\right)\right|. \end{array}$$

and when $f_{d}=0$, the range ambiguity function is obtained as

(86) $$\left|\chi (\tau ,0)\right|=\left\{\begin{array}{cc} \left|\sum_{m,n,p,q}U\left(m,n,p,q,\tau ,0\right)\right|, & |\tau |<N_{s}T,\\ 0, & \mathrm{others}, \end{array}\right.$$

From the subsequent simulations, it can be observed that OCDM, like OFDM, is sensitive to both delay and Doppler effects, making it suitable for sensing applications.

## 5. OCDM-Based Sensing Detection Performance Analysis

Consider a transmitter employing echoes for target detection. When using the signal in (6) for transmission, two hypotheses are defined: $H_{0}$ and $H_{1}$. $H_{0}$ corresponds to the scenario where the received echo signals consist solely of noise, indicating the absence of targets. In contrast, $H_{1}$ denotes the presence of echoes from the transmitted signal, implying the existence of one or more targets. These hypotheses can be formally expressed as:

(87) $$\begin{array}{c} H_{0}:r=n,\;H_{1}:r=\mathrm{Hs}+n, \end{array}$$

where r represents the received echo signal, and H represents the cyclic matrix containing the channel impulse response, consistent with (7).

### 5.1. MF Detector Performance Analysis

Matched-filter detection is implemented by correlating the received signal with a locally stored reference sequence whose autocorrelation properties are optimized for sensing. The reference is a known training symbol that is appended to the transmitted waveform. Correlation peaks are then compared with a threshold that is set adaptively via a CFAR procedure to guarantee a prescribed probability of false alarm.

Let the detection threshold be denoted by $V_{T}$, and define the false alarm probability as the probability that the correlation peak exceeds this threshold under the assumption $H_{0}$. Let the local reference sequence be u. Then, the matched filter output under $H_{0}$ can be expressed as:

(88) $$\begin{array}{c} R=\sum_{k=0}^{N-1}u^{*}\left(k\right)n\left(k\right), \end{array}$$

where *N* represents the number of OCDM carriers.

Let uRe(k)=Re(u(k)), uIm(k)=Im(u(k)), nRe(k)=Re(n(k)), nIm(k)=Im(n(k)). Furthermore, define

(89) RRe=∑k=0N−1uRe(k)nRe(k)RIm=∑k=0N−1uIm(k)nIm(k)nRe2=∑k=0N−1(uRe(k))2nIm2=∑k=0N−1(uIm(k))2.

With these definitions, the above equation can be rewritten as

(90) ∑k=0N−1u*(k)n(k)=∑k=0N−1uRe(k)−juIm(k)nRe(k)+jnIm(k)=∑k=0N−1uRe(k)nRe(k)+∑k=0N−1uIm(k)nIm(k)+j∑k=0N−1uRe(k)nIm(k)−∑k=0N−1uIm(k)nRe(k).

Let

(91) RI=∑k=0N−1uRe(k)nRe(k)+∑k=0N−1uIm(k)nIm(k)RQ=∑k=0N−1uRe(k)nIm(k)+∑k=0N−1uIm(k)nRe(k).

Since each element of nRe(k) and nIm(k) is independently distributed as $N\left(0,\sigma^{2}/2\right)$, it follows that RRe∼N0,σ2/(2nRe2) and RIm∼N0,σ2/(2nIm2). Assuming the local sequence amplitude is constant and normalized such that (uRe(k))2+(uIm(k))2=1, then $R_{I}∼N\left(0,N\sigma^{2}/2\right)$, and similarly for $R_{Q}$. Therefore, the Probability Density Function (PDF) of $\left|R\right|=\sqrt{R_{I}^{2}+R_{Q}^{2}}$ is:

(92) $$\begin{array}{c} f\left(R|H_{0}\right)=\frac{2R}{N\sigma^{2}}e^{-\frac{R^{2}}{N\sigma^{2}}}. \end{array}$$

Thus, the false alarm probability is

(93) $$\begin{array}{cc} P_{f} & =\int_{V_{T}}^{+\infty }f(R|H_{0})dR=\int_{V_{T}}^{+\infty }\frac{2R}{N\sigma^{2}}e^{-\frac{R^{2}}{N\sigma^{2}}}dR=-e^{-\frac{R^{2}}{N\sigma^{2}}}{|}_{V_{T}}^{+\infty }=e^{-\frac{{V_{T}}^{2}}{N\sigma^{2}}}. \end{array}$$

Given a false alarm probability $P_{f}$, the detection threshold should be set as follows:

(94) $$\begin{array}{c} V_{T}=\sqrt{-N\sigma^{2}\ln P_{f}}. \end{array}$$

Although the transmitted signal possesses favorable autocorrelation properties, these characteristics are inevitably degraded by multipath channel effects. Without loss of generality, it is assumed that the first path of the multipath channel experiences the least attenuation, enabling the extraction of a sequence with good autocorrelation from the received signal corresponding to this path. To mitigate the influence of subsequent paths, and leveraging the orthogonality and cyclic properties of $\Phi^{H}$, this paper proposes the following form for the training symbol.

Let the training symbol x satisfy $x={\left[\sqrt{N},0,0,\ldots ,0\right]}^{T}$, and ${\left[\Phi^{H}\right]}_{:,k}$ represents the *k*-th column of $\Phi^{H}$. From (6), it follows that the transmitted signal s is $\sqrt{N}$ times the first column of $\Phi^{H}$, i.e., $s=\sqrt{N}{\left[\Phi^{H}\right]}_{:,1}$. Therefore, $H_{1}$ can be rewritten as:

(95) $$\begin{array}{c} r=\mathrm{Hs}+n=\mathrm{Sh}+n. \end{array}$$

where S is a cyclic matrix with s as the first column, so $S=\sqrt{N}\Phi^{H}$. The received signal r can be expressed as a weighted linear combination of the column vectors of S, that is

(96) $$\begin{array}{c} r=\sqrt{N}\Phi^{H}h+n=\sqrt{N}\sum_{k=1}^{K}h_{k}{\left[\Phi^{H}\right]}_{:,k}+n. \end{array}$$

At the receiver, the local sequences $u=s=\sqrt{N}{\left[\Phi^{H}\right]}_{:,1}$ are correlated with the received signal r. Since the columns of $\Phi^{H}$ are mutually orthogonal, interference from other channel components does not affect the correlation outcome. The resulting correlation peak is given by

(97) $$\begin{array}{cc} R & =\sum_{k=0}^{N-1}u^{*}\left(k\right)r\left(k\right)=\sum_{k=0}^{N-1}N{\left[\Phi^{T}\right]}_{k,1}⊙\left(\sum_{j=1}^{K}h_{j}{\left[\Phi^{H}\right]}_{k,j}+n\left(k\right)\right). \end{array}$$

where ⊙ denotes the Hadamard product. Based on the analysis in (91) and (97), it follows that RI∼NRe(h1)N,Nσ2/2 and RQ∼NIm(h1)N,Nσ2/2. Accordingly, the PDF of |R| is

(98) $$\begin{array}{c} f\left(R|H_{1}\right)=\frac{2R}{N\sigma^{2}}e^{-\frac{R^{2}+{\left|h_{1}\right|}^{2}N^{2}}{N\sigma^{2}}}I_{0}\left(\frac{2\left|h_{1}\right|R}{\sigma^{2}}\right). \end{array}$$

where $I_{0}\left(x\right)=1/2\pi \int_{0}^{2\pi }e^{x\cos\varphi }d\varphi$ denotes the modified Bessel function of the first kind and zero order.

Therefore, the probability of detection is

(99) $$\begin{array}{cc} P_{d} & =\int_{V_{T}}^{+\infty }f(R|H_{1})dR=\int_{V_{T}}^{+\infty }\frac{2R}{N\sigma^{2}}e^{-\frac{R^{2}+{\left|h_{1}\right|}^{2}N^{2}}{N\sigma^{2}}}I_{0}\left(\frac{2\left|h_{1}\right|R}{\sigma^{2}}\right)dR. \end{array}$$

Let $x=\sqrt{2/N\sigma^{2}}R$ and $a=\sqrt{2|h_{1}{|}^{2}N/\sigma^{2}}$; then $R=\sqrt{N\sigma^{2}/2}x$ and $dR=\sqrt{N\sigma^{2}/2}dx$. Accordingly, the above equation can be rewritten as:

(100) $$\begin{array}{cc} P_{d} & =\int_{V_{T}}^{+\infty }\frac{2R}{N\sigma^{2}}e^{-\frac{1}{2}\left(\frac{2R^{2}}{N\sigma^{2}}+\frac{2{\left|h_{1}\right|}^{2}}{\sigma^{2}}\right)}I_{0}\left(\frac{2\left|h_{1}\right|R}{\sigma^{2}}\right)dR\\ \; & =\int_{\sqrt{\frac{2}{N\sigma^{2}}}V_{T}}^{+\infty }xe^{-\frac{x^{2}+a^{2}}{2}}I_{0}\left(ax\right)dx=q\left(a,b\right). \end{array}$$

where $b=\sqrt{2/N\sigma^{2}}V_{T}$, and

(101) $$\begin{array}{c} q\left(a,b\right)=\int_{b}^{+\infty }xe^{-\frac{x^{2}+a^{2}}{2}}I_{0}\left(ax\right)dx. \end{array}$$

denotes the Marcum Q function.

The computational complexity of the MF detector arises from the sequence multiplications required to evaluate the correlation peak in (97), leading to an overall complexity of O(N). The MF is attractive for high-speed systems due to its low complexity and efficient implementation via correlation operations or FFT-based methods. Its feasibility lies in requiring minimal channel knowledge, making it suitable when CSI is inaccurate under rapid mobility [35,36].

Under fading channels, the performance of the MF detector is affected by multipath propagation and channel fluctuations. Multipath fading can reduce the peak amplitude of the correlation output or generate multiple interfering peaks, increasing the probability of false alarms and missed detections. When a strong Line-of-Sight path exists, the MF detector can maintain relatively robust performance, whereas in purely scattered or fast-fading environments, its detection performance degrades significantly. Performance can be improved by employing techniques such as multipath energy combining or adaptive thresholding to enhance robustness against fading channels [36].

### 5.2. GLRT Detector Performance Analysis

As analyzed in the previous section, the performance of MF is susceptible to multipath effects, making it challenging to achieve reliable detection in severe multipath fading environments. To address this issue, this section investigates the detection performance of OCDM based on the GLRT. The likelihood ratio quantifies the probability that the received signal consists of signal-plus-noise versus noise alone. When the likelihood ratio exceeds a predefined threshold, there is sufficient evidence to support the presence of a signal. According to [22], the GLRT for the received signal r is defined as:

(102) $$\begin{array}{c} L_{G}\left(r\right)=\frac{f(r;\hat{h},H_{1})}{f(r;H_{0})}, \end{array}$$

where $f(r;\hat{h},H_{1})$ denotes the PDF of the received signal under hypothesis $H_{1}$, where $\hat{h}$ is the estimated channel impulse response. Similarly, $f(r;H_{0})$ denotes the PDF of the received signal under hypothesis $H_{0}$. Based on (87), we obtain:

(103) $$\begin{array}{c} f\left(r;H_{0}\right)=\frac{1}{{\left(\pi \sigma^{2}\right)}^{N}}e^{-\frac{{∥r∥}^{2}}{\sigma^{2}}} \end{array}$$

and

(104) $$\begin{array}{c} f\left(r;\hat{h},H_{1}\right)=\frac{1}{{\left(\pi \sigma^{2}\right)}^{N}}e^{-\frac{{∥r-S\hat{h}∥}^{2}}{\sigma^{2}}}. \end{array}$$

Therefore, taking the natural logarithm of (102) yields:

(105) $$\begin{array}{cc} \ln L_{G}\left(r\right) & =-\frac{1}{\sigma^{2}}\left({∥r-S\hat{h}∥}^{2}-{∥r∥}^{2}\right)=-\frac{1}{\sigma^{2}}\left[{\left(r-S\hat{h}\right)}^{H}\left(r-S\hat{h}\right)-r^{H}r\right]\\ \; & =\frac{1}{\sigma^{2}}\left(r^{H}S\hat{h}+{\hat{h}}^{H}S^{H}r-{\hat{h}}^{H}S^{H}S\hat{h}\right). \end{array}$$

Substituting $\hat{h}={\left(S^{H}S\right)}^{-1}S^{H}r$ into the above equation yields:

(106) $$\begin{array}{c} \ln L_{G}\left(r\right)=\frac{1}{\sigma^{2}}\left[r^{H}S{\left(S^{H}S\right)}^{-1}S^{H}r\right]. \end{array}$$

By designing the training symbols such that S is full rank, the above equation simplifies to:

(107) $$\begin{array}{c} \ln L_{G}\left(r\right)=\frac{r^{H}r}{\sigma^{2}}. \end{array}$$

Therefore, assuming that $\ln L_{G}\left(r\right)=n^{H}n/\sigma^{2}∼\chi^{2}\left(N\right)$ holds under hypothesis $H_{0}$, and letting $z=\ln L_{G}\left(r\right)$, the corresponding PDF is given by:

(108) $$\begin{array}{c} f\left(z\right)=\frac{1}{2\sigma^{2}\Gamma \left(N/2\right)}{\left(\frac{z}{2\sigma^{2}}\right)}^{N/2-1}e^{-\frac{z}{2\sigma^{2}}}. \end{array}$$

Therefore, the false alarm probability is [37]:

(109) $$\begin{array}{c} P_{f}=\int_{V_{T}}^{+\infty }f(z)dz=e^{-\frac{V_{T}}{2\sigma^{2}}}\sum_{i=0}^{N/2-1}\frac{1}{i!}{\left(\frac{V_{T}}{2\sigma^{2}}\right)}^{i}. \end{array}$$

Given a predefined false alarm probability, the corresponding detection threshold can be determined by numerically evaluating the above equation.

The computational complexity of the GLRT detector originates from the evaluation of the maximum likelihood function in (107), which, in this case, reduces to computing the correlation of the received sequence. Therefore, the overall complexity is O(N). This is consistent with the ZF. However, for MIMO systems, the implementation of the GLRT requires maximization over unknown parameters, which results in considerably higher computational complexity. Therefore, practical high-speed systems often resort to approximate or low-complexity GLRT variants, such as subspace projection or coarse grid search, which can substantially reduce the computational burden while maintaining reliable detection performance [38,39,40].

The GLRT detector estimates the channel gain using a maximum likelihood approach, allowing it to effectively compensate for channel variations under slow-fading conditions, thereby exhibiting high detection robustness. However, in fast-fading scenarios, channel estimation errors may introduce bias in the test statistic, leading to increased false alarm or missed detection rates. To mitigate these effects, advanced channel estimation or prediction methods can be incorporated to enhance GLRT performance in dynamic fading environments [41,42].

## 6. Simulation Results

This section presents numerical results that simultaneously assess the communication and sensing performance of OCDM. BER curves are generated over the Extended Vehicular A (EVA) channel and a Linear Time-Variant (LTV) channel with a mobile speed of 300 km/h; QPSK is used for the LTV scenario. All BER curves have been annotated with a horizontal Forward Error Correction (FEC) line at $3.8\times 10^{-3}$. OFDM, OCDM, and SC-FDE are compared under ZF, MMSE, MLE, and IB-DFE equalization for multiple modulation orders. The ambiguity function and detection performance are examined via MF and GLRT detectors, with OFDM serving as the benchmark; neither OFDM nor OCDM embed carrier information. Additional parameters are listed in Table 3 and Table 4.

**Figure 2:**
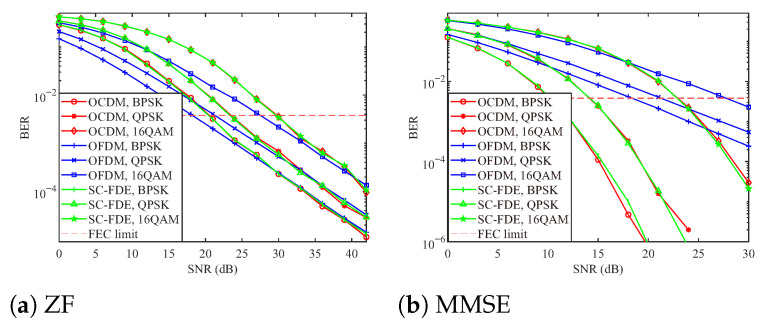
BER performance of three systems under linear equalization under different modulation schemes.

The BER performance curves of the OCDM, OFDM, and SC-FDE systems under linear equalization for three modulation schemes—BPSK, QPSK, and 16QAM—are illustrated in Figure 2. As observed from the figure, the BER performance of the OCDM and SC-FDE systems under ZF equalization is nearly identical. For all three modulation schemes, the two precoded systems underperform OFDM at low SNR but outperform it at high SNR. Moreover, the SNR at which the BER curves intersect increases with the modulation order. Under MMSE equalization, the BER performance of the OCDM and SC-FDE systems remains closely aligned. For BPSK and QPSK modulation, both systems outperform OFDM across the entire SNR range. However, for 16QAM modulation, their performance is inferior to that of OFDM at low SNR and superior at high SNR. These simulation results are in strong agreement with the theoretical analysis. It is noteworthy that OCDM with BPSK under MMSE detection reaches the FEC limit at approximately 12 dB, which is about 5 dB better than ZF. This improvement can provide enhanced error-correction redundancy.

**Figure 3:**
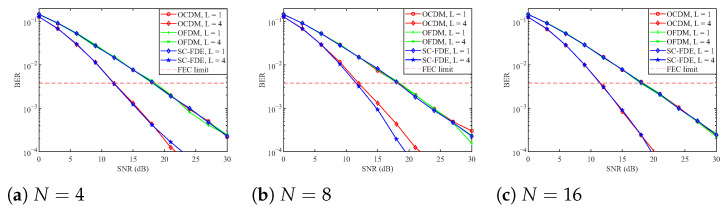
BER performance curves of three systems under MLE.

Figure 3 presents the BER performance curves of the three systems under MLE [43], evaluated over single-path and four-path channels. As shown in the figure, the BER performance of all three systems is essentially identical in the single-path scenario, since the achievable multipath diversity gain is 1 in all cases. However, the OCDM and SC-FDE systems exhibit significantly better BER performance than OFDM, as both systems benefit from a multipath diversity gain greater than 1. Moreover, when the subcarrier count is increased from N=4 to N=8, or even higher, OCDM and SC-FDE exhibit a marked BER improvement, since the likelihood that L=1 in (65) diminishes with *N*, thereby enlarging the attainable diversity gain. Indeed, for N≥16 the multipath diversity of OCDM under MLE approaches the total number of available propagation paths. Moreover, it can be observed that under the four-path channel, all considered systems reach the FEC limit at approximately 12 dB, which is significantly better than linear equalization.

**Figure 4:**
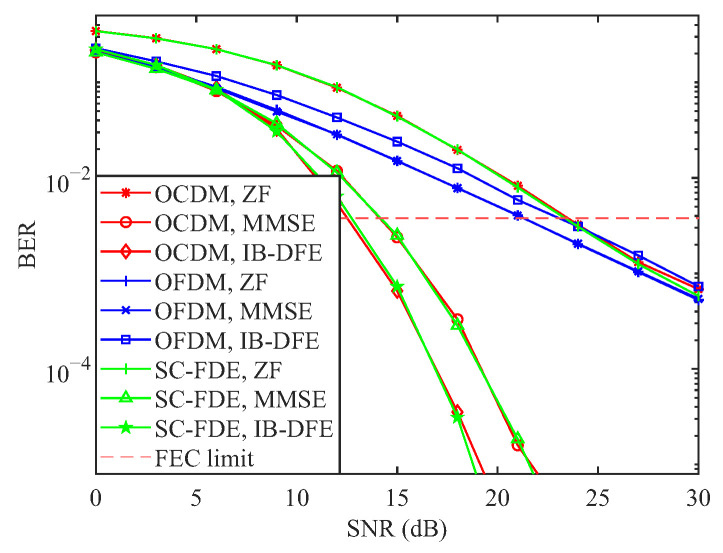
BER performance of three systems with different equalisation modes at 0 km/h.

Figure 4 illustrates the BER performance curves of the three systems under three equalization schemes: ZF, MMSE, and IB-DFE. As shown in the figure, the BER performance of the OFDM system remains nearly identical under both ZF and MMSE equalization. In contrast, the BER performance of the OCDM and SC-FDE systems is further improved under IB-DFE compared to MMSE equalization. However, for the OFDM system, due to its relatively poor BER performance under MMSE equalization, error propagation arises during the feedback process, leading to a degradation in performance under IB-DFE relative to MMSE. In contrast, for systems such as SC-FDE and OCDM, which exhibit multipath diversity gains, the use of nonlinear equalization techniques like IB-DFE significantly enhances error performance. The figure further illustrates that IB-DFE attains the FEC limit earlier than other linear equalization schemes, exhibiting performance comparable to that of MLE, thereby highlighting its superior error-correction capability among linear methods.

**Figure 5:**
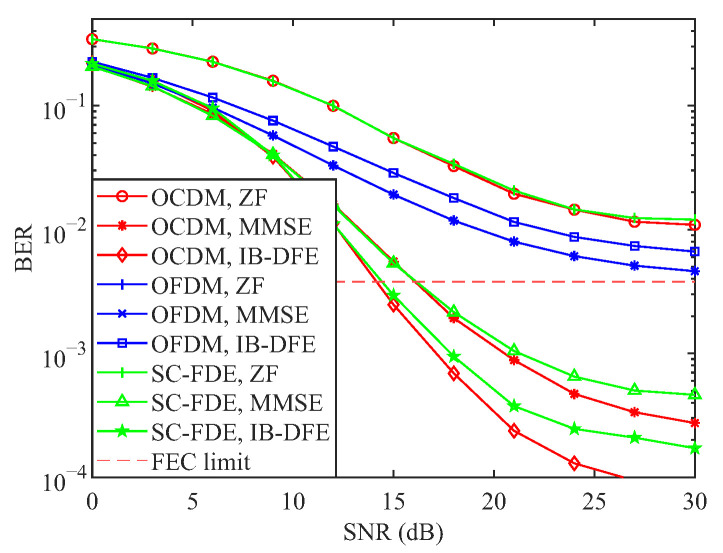
BER performance of three systems with different equalization modes at a speed of 300 km/h.

Figure 5 presents the BER performance comparison of OCDM, OFDM, and SC-FDE under ZF and MMSE equalization for QPSK modulation at a mobile station speed of 300 km/h. As shown, the BER performance of the SC-FDE and OCDM systems under ZF equalization is inferior to that of the OFDM system but surpasses OFDM performance under MMSE equalization. Moreover, the OCDM system with MMSE equalization outperforms the SC-FDE system at high SNR in the presence of Inter-Carrier Interference (ICI). OCDM systems employing MMSE and IB-DFE equalization can both reach the FEC limit at approximately 15 dB, whereas OFDM fails to achieve it. This advantage arises because each OCDM carrier occupies a larger channel bandwidth, thereby dispersing the ICI over a wider spectrum and enhancing ICI resilience. Consequently, OCDM demonstrates superior communication capability in complex channel environments.

**Figure 6:**
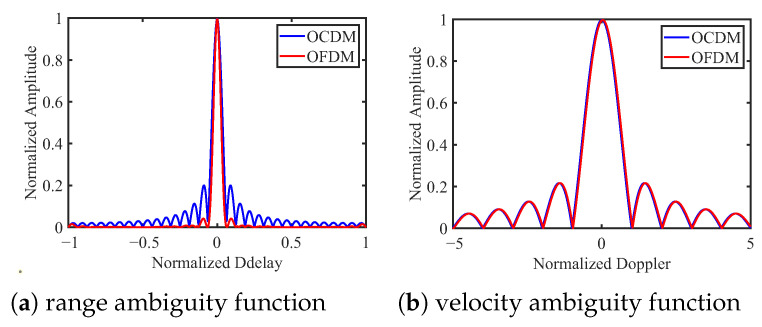
Comparison of OCDM and OFDM ambiguity functions.

Figure 6 presents the range and velocity ambiguity functions for both the OCDM and OFDM waveforms. It can be observed that, regarding the range ambiguity function, the OCDM waveform exhibits a steep roll-off comparable to that of OFDM, along with a low sidelobe level below 0.2, indicating good range resolution. For the velocity ambiguity function, the performances of both waveforms are comparable, demonstrating that the OCDM waveform maintains strong sensitivity to Doppler shifts. These results indicate that the OCDM waveform is well-suited for sensing applications.

**Figure 7:**
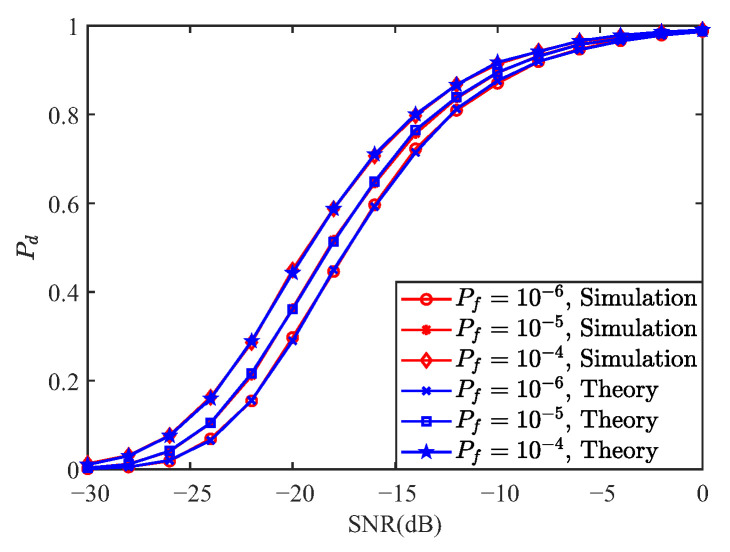
Theoretical and simulation comparison of MF-based OCDM waveform detection probability.

Figure 7 presents a comparison between the theoretical and simulated detection probabilities of OCDM under matched filter detection for false alarm probabilities of $10^{-6}$, $10^{-5}$, and $10^{-4}$, respectively. It can be observed that the theoretical and simulation curves exhibit excellent agreement, thereby validating the correctness of the theoretical derivation. Furthermore, the OCDM-ISAC waveform employing the matched filter achieves a detection probability exceeding 95% at −5 dB, demonstrating the effectiveness of OCDM for sensing applications.

**Figure 8:**
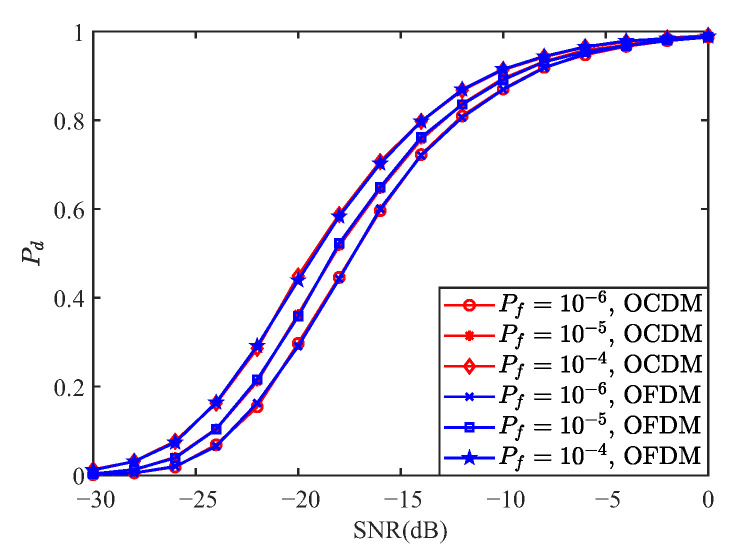
Comparison of detection probabilities between MF-based OCDM and OFDM waveforms.

Figure 8 presents the comparison of the detection performance of OCDM and OFDM under the MF detector for false alarm probabilities of $10^{-6}$, $10^{-5}$, and $10^{-4}$. As shown, both waveforms exhibit nearly identical performance. From (97), the OCDM transmitted signal corresponds to the first column of $F^{H}$, which consists entirely of 1 s, making S an all-ones matrix. Considering multipath effects, if the phases of the other paths align with the first path, they enhance its correlation peak; otherwise, they weaken it. Given that the energy of the non-dominant paths is small and assuming equal probability of constructive or destructive interference, the detection performance of OFDM and OCDM can be considered equivalent, as confirmed by the simulation results in Figure 8.

**Figure 9:**
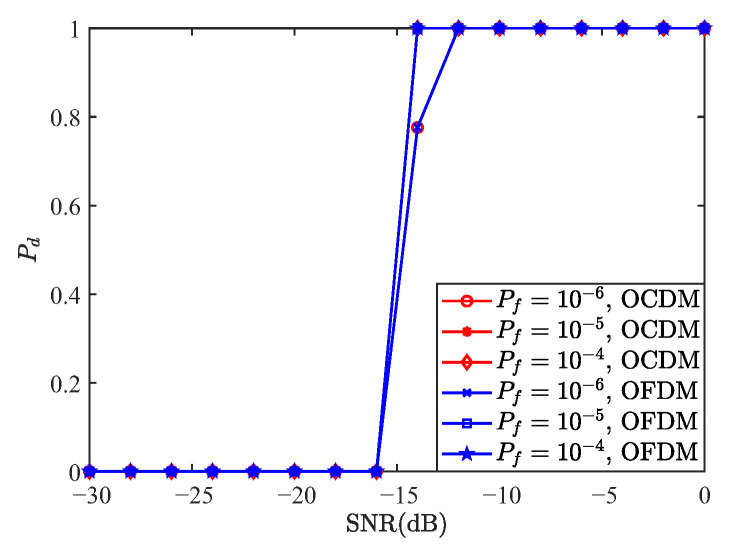
Comparison of detection probabilities between GLRT-based OCDM and OFDM waveforms.

Figure 9 compares the detection performance of OCDM and OFDM waveforms under the GLRT detector for false alarm probabilities of $10^{-6}$, $10^{-5}$, and $10^{-4}$. As observed from the figure, both waveforms exhibit nearly identical detection performance under the GLRT detector. This consistency can be explained by Equation (107), which shows that the likelihood ratio depends solely on the received signal power and the noise power. Given that the transmitted signal power is identical for both OCDM and OFDM waveforms, their expected received signal power is also identical. Consequently, the detection probabilities of the two waveforms under the GLRT detector are essentially the same.

**Figure 10:**
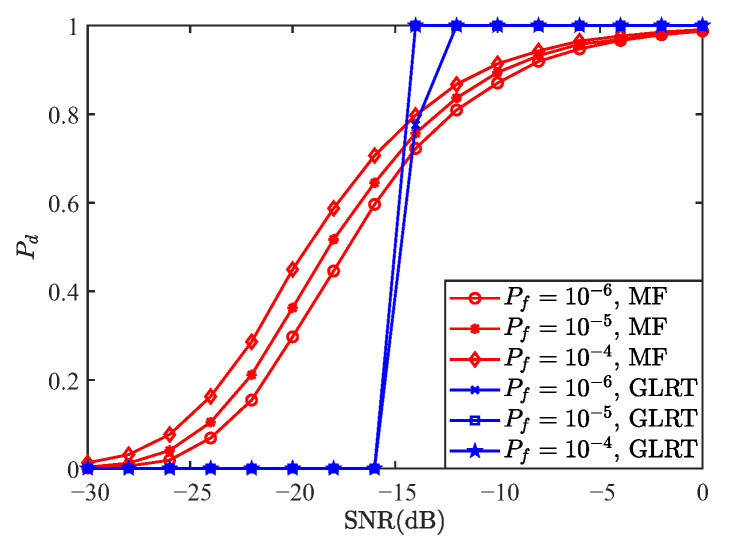
Detection performance comparison of OCDM waveforms based on MF and GLRT detector.

Figure 10 compares the detection performance of the OCDM waveform under the GLRT and MF detectors for false alarm probabilities of $10^{-6}$, $10^{-5}$, and $10^{-4}$. As illustrated, the GLRT detector exhibits inferior performance to the MF detector at low SNRs, whereas it outperforms the MF detector at high SNRs. This behavior arises from the fundamental nature of the GLRT detector, which is based on the received signal power. At low SNRs, the high noise power necessitates large detection thresholds to maintain a constant false alarm rate, resulting in diminished sensitivity to target echoes. Conversely, at high SNRs, the thresholds become lower relative to the signal power, enhancing detection capability. Consequently, GLRT detection underperforms MF detection at low SNRs but surpasses it at higher SNRs. Nevertheless, as shown in the figure, for false alarm probabilities no greater than $10^{-5}$, the GLRT detector achieves nearly 100% detection probability when the SNR exceeds −14 dB, demonstrating its strong effectiveness in high-SNR regimes.

## 7. Conclusions

This paper presents a preliminary investigation into the feasibility and effectiveness of employing OCDM waveforms in ISAC systems under complex scenarios. First, the BER performance of OCDM is analyzed under various equalization schemes. Then, the ambiguity function of the OCDM waveform is analytically derived and simulated to demonstrate its potential in sensing and detection applications. Subsequently, the detection performance of OCDM waveforms is evaluated under both MF and GLRT detectors. A closed-form expression for the detection probability under MF detection is rigorously derived. Simulation results validate the superior communication performance of OCDM waveforms in challenging environments, their reliability in sensing tasks, and the accuracy of the theoretical detection expression, thereby confirming the overall effectiveness of OCDM in ISAC systems.

It is important to emphasize that this study represents a preliminary investigation into the applicability of OCDM waveforms in complex ISAC scenarios. Several critical aspects remain unaddressed and will be the focus of future research. First, comprehensive parameter estimation—including velocity, range, azimuth, and elevation angle—has not been explored; developing accurate estimation algorithms for these parameters is essential to fully leverage OCDM waveforms for practical sensing applications. Second, the detection performance under general channel conditions, particularly when fading coefficients follow a complex Gaussian distribution, has not been analytically characterized. Establishing theoretical expressions for detection probability under such conditions will provide rigorous performance benchmarks and guide waveform optimization. Addressing these gaps is crucial for validating the practical utility of OCDM-based ISAC systems and for advancing their deployment in realistic wireless environments.

## Figures and Tables

**Table 1:** List of abbreviations.

5G	Fifth-Generation
6G	Sixth-Generation
BER	Bit Error Rate
BPSK	Binary Phase-Shift Keying
CFAR	Constant False Alarm Rate
CP	Cyclic Prefix
DFnT	Discrete Fresnel Transform
DFT	Discrete Fourier Transform
EVA	Extended Vehicular A
FEC	Forward Error Correction
FFT	Fast Fourier Transform
GLRT	Generalized Likelihood Ratio Test
IB-DFE	Iterative Block-Decision Feedback Equalization
ICI	Inter-Carrier Interference
IDFnT	Inverse Discrete Fresnel Transform
IDFT	Inverse Discrete Fourier Transform
IoT	Internet of Things
IoV	Internet of Vehicles
ISAC	Integrated Sensing and Communication
LTI	Linear Time-Invariant
LTV	Linear Time-Variant
MF	Matched Filter
MIMO	Multiple Input Multiple Output
MLE	Maximum Likelihood Equalization
MMSE	Minimum Mean Square Error
mMTC	Massive Machine-Type Communication
OCDM	Orthogonal Chirp Division Multiplexing
OFDM	Orthogonal Frequency Division Multiplexing
PDF	Probability Density Function
QAM	Quadrature Amplitude Modulation
QPSK	Quadrature Phase-Shift Keying
SC-FDE	Single-Carrier Frequency-Domain Equalization
SNR	Signal-to-Noise Ratio
uRLLC	Ultra-Reliable and Low-Latency Communication
ZC	Zadoff–Chu
ZF	Zero Forcing

**Table 2:** Complexity of equalization methods.

Methods	OFDM	SC-FDE	OCDM
ZF	O(NlogN)	O(NlogN)	O(NlogN)
MMSE	O(NlogN)	O(NlogN)	O(NlogN)
MLE	O(NM)	$O(N^{N}N^{2})$	$O(N^{3})$
IB-DFE	$O(N^{2})$	$O(N^{2})$	$O(N^{2})$

**Table 3:** Simulation parameters.

Simulation	Simulation Type	
Parameters	Communication	Sensing
Channel Model	EVA, LTV	EVA
Frequency of Carrier	2 GHz	2 GHz
OCDM Symbol Period	1 μ s	1μ s
OFDM Subcarrier Interval	1 MHz	1 MHz
Number of Symbols per Subframe	14	14
Number of Subcarriers	16	16
Cyclic Prefix Length	$L_{m}+1$	$L_{m}+1$

**Table 4:** EVA channel parameters.

Multipath	Delay (ns)	Average Path Gain (dB)
1	0	0
2	30	−1.5
3	150	−1.4
4	310	−3.6
5	370	−0.6
6	710	−9.1
7	1090	−7.0
8	1730	−12.0
9	2510	−16.9

## Data Availability

The original contributions presented in this study are included in the article. Further inquiries can be directed to the corresponding authors.
